# Differential proteomics argues against a general role for CD9, CD81 or CD63 in the sorting of proteins into extracellular vesicles

**DOI:** 10.1002/jev2.12352

**Published:** 2023-07-31

**Authors:** Yé Fan, Cédric Pionneau, Federico Cocozza, Pierre‐Yves Boëlle, Solenne Chardonnet, Stéphanie Charrin, Clotilde Théry, Pascale Zimmermann, Eric Rubinstein

**Affiliations:** ^1^ Centre d'Immunologie et des Maladies Infectieuses Sorbonne Université, Inserm, CNRS Paris France; ^2^ UMS Production et Analyse des données en Sciences de la vie et en Santé, PASS Plateforme Post‐génomique de la Pitié‐Salpêtrière, P3S Sorbonne Université, Inserm Paris France; ^3^ Inserm U932, Institut Curie Centre de Recherche PSL Research University Paris France; ^4^ Institut Pierre Louis d’Épidémiologie et de Santé Publique Sorbonne Université, Inserm Paris France; ^5^ CurieCoretech Extracellular Vesicles Institut Curie Centre de Recherche Paris France; ^6^ Centre de Recherche en Cancérologie de Marseille (CRCM) Institut Paoli‐Calmettes, Aix‐Marseille Université, Inserm, CNRS Marseille France; ^7^ Department of Human Genetics Katholieke Universiteit Leuven (KU Leuven) Leuven Belgium

**Keywords:** CD63, CD81, CD9, exosomes, extracellular vesicles, IgSF8, mass spectrometry, proteomic, PTGFRN, tetraspanins

## Abstract

The tetraspanins CD9, CD81 and CD63 are major components of extracellular vesicles (EVs). Yet, their impact on EV composition remains under‐investigated. In the MCF7 breast cancer cell line CD63 was as expected predominantly intracellular. In contrast CD9 and CD81 strongly colocalized at the plasma membrane, albeit with different ratios at different sites, which may explain a higher enrichment of CD81 in EVs. Absence of these tetraspanins had little impact on the EV protein composition as analysed by quantitative mass spectrometry. We also analysed the effect of concomitant knock‐out of CD9 and CD81 because these two tetraspanins play similar roles in several cellular processes and associate directly with two Ig domain proteins, CD9P‐1/EWI‐F/PTGFRN and EWI‐2/IGSF8. These were the sole proteins significantly decreased in the EVs of double CD9‐ and CD81‐deficient cells. In the case of EWI‐2, this is primarily a consequence of a decreased cell expression level. In conclusion, this study shows that CD9, CD81 and CD63, commonly used as EV protein markers, play a marginal role in determining the protein composition of EVs released by MCF7 cells and highlights a regulation of the expression level and/or trafficking of CD9P‐1 and EWI‐2 by CD9 and CD81.

## 1 INTRODUCTION

Many prokaryotic and eukaryotic cells secrete vesicles limited by a lipid bilayer. These extracellular vesicles (EVs) are now considered as important mediators of intercellular communication, involved in both physiological and pathological processes (Yáñez‐Mó et al., 2015) including immune responses (Lindenbergh & Stoorvogel, 2018) and tumour progression (Kalluri & LeBleu, 2020; Tkach & Théry, 2016).

The classification of EVs is based on their size and their biogenesis (van Niel et al., 2018). EVs released by parental cells through outward budding of the plasma membrane, have a diameter comprised between 50 nm to over 1 μm and are termed microvesicles or ectosomes. Others, known as exosomes, have an endosomal origin, and are released by exocytosis via the fusion of multivesicular bodies (MVBs) with the plasma membrane. Exosomes are small vesicles with a diameter smaller than 150 nm and are enriched in endosome‐derived components. However, when the sub‐cellular origin of the analysed EVs is not known, which is often the case when EVs are recovered from the extracellular medium without accompanying intracellular tracking analyses, a conservative nomenclature we will implement in this article is to use the generic term EVs (Witwer & Théry, 2019).

The composition of EVs is dependent on their cellular origin, the physiological state of the cell that secretes them as well as the mechanisms of their biogenesis (Tkach & Théry, 2016; van Niel et al., 2018; Yáñez‐Mó et al., 2015). EVs contain soluble cytosolic proteins, nucleic acids, lipids as well a number of transmembrane proteins. Among the latter, three tetraspanin family members, CD9, CD63 and CD81, are widely used markers of EV subsets and are used as targets to immuno‐isolate EVs.

Tetraspanins form a superfamily of proteins characterized by four transmembrane domains and a specific fold in the largest of the two extracellular domains. All cell types and tissues express a variable combination of tetraspanins, some of which are mainly present at the plasma membrane, such as CD9, or in contrast intracellular, such as CD63, which is enriched in late endosomes and lysosome‐related organelles (Charrin et al., 2014; Charrin, Le Naour et al., 2009; Hemler, 2005). Tetraspanins have been involved in a wide range of cellular functions and their deficiencies, either in mice or in humans, sometimes yield a dramatic effect. At the molecular level, tetraspanins regulate the trafficking, membrane compartmentalization and the function of their direct molecular partners (Charrin et al., 2014; Charrin, Le Naour et al., 2009; Hemler, 2005). For example, CD81 deficiencies in mice and humans result in impaired humoral response due to the decreased expression of CD19, a key B‐cell costimulatory molecule (Levy, 2014). CD63 KO mice have impaired pigmentation due to an alteration of the trafficking and processing of pmel17, a molecule involved in melanogenesis, as well as a renal phenotype including polyuria with a parallel decrease in urine osmolality, and mislocalisation of the organic cation transporter OCT2 (Schröder et al., 2009; Schulze et al., 2017; van Niel et al., 2011). Importantly, CD9 and CD81 are closely related tetraspanins, sharing 45% identity, and play similar roles in cell fusion processes (Charrin et al., 2014; Charrin, Le Naour et al., 2009; Hemler, 2005). They both play an essential role in sperm‐egg fusion (Le Naour et al., 2000; Rubinstein et al., 2006), but negatively regulate HIV‐induced syncytium formation as well as macrophage and muscle cell fusion (Charrin et al., 2013; Takeda et al., 2003; Weng et al., 2009). CD9 and CD81 both associate directly with two related Ig domain proteins, CD9P‐1/EWI‐F (encoded by the *PTGFRN* gene) and EWI‐2 (encoded by the *IGSF8* gene) (Charrin et al., 2003, 2001; Stipp, Kolesnikova et al., 2001; Stipp, Orlicky et al., 2001), which also regulate several fusion processes (Charrin et al., 2013; Cohen et al., 2022; Whitaker et al., 2019).

The roles of CD9, CD81 and CD63 in EV biogenesis and function are poorly understood. It has been suggested that tetraspanins could play a role in EV targeting and uptake, or act as sorting machineries towards exosomes (Andreu & Yanez‐Mo, 2014). A recent study found no evidence for CD9 and CD63 playing a role in the EV content delivery process (Tognoli et al., 2023). To address the second hypothesis, we analysed by mass‐spectrometry the protein composition of EVs secreted by MCF7 cells, lacking or not the main tetraspanins used for EV characterization, CD9, CD81 and CD63.

## 2 MATERIAL AND METHODS

### 2.1 Cell culture and generation of cells lacking tetraspanins

MCF7 cells, derived from a breast adenocarcinoma patient, were initially obtained from ATCC, and validated by STR in 2018. They were cultured in Dulbecco's modified Eagle's medium supplemented with 10% foetal calf serum (Sigma) and antibiotics (penicillin/streptomycin, Eurobio). MCF7 KO for CD9, CD81 and CD63 were generated using the CRISPR/ Cas9 gene editing technology. Target sequences (CD9: TTGGACTATGGCTCCGATTC; CD81: AGGAATCCCAGTGCCTGCTG; CD63: CTGAGTCAGACCATAATCCA) were selected using the CRISPR design tool available at the Broad Institute (https://portals.broadinstitute.org/gpp/public/analysis‐tools/sgrna‐design). The corresponding guide DNA sequences were cloned into the lentiCRISPRv2 plasmid (Addgene #52961) according to the instructions of the Zhang laboratory (Sanjana et al., 2014). The plasmids were transfected using Fugene HD (Promega) according to the manufacturer's instructions, and cells were treated after 36−48 h with 5 μg/mL puromycin for 24 h. After staining with CD9, CD81 or CD63 mAbs, cells that had lost the expression of the targeted tetraspanin were sorted using a FACS Aria cell sorter (Becton Dickinson).

### 2.2 Monoclonal antibodies and plasmids

All mAbs used directed to human CD9, CD81 and CD63, CD9P‐1, EWI‐2 or the integrin β1 subunit (respectively TS9, TS81, TS63, 1F11, 8A12 and β1‐vjf, in‐house made) have been previously described (Charrin et al., 2003, 2001). The plasmid encoding EWI‐2 was previously described (Charrin et al., 2003) and that encoding mCherry‐Sec61β was a gift from Gia Voeltz (Addgene plasmid catalogue no. 49155) (Zurek et al., 2011).

### 2.3 Flow‐cytometry analysis

Cells were detached with a non‐enzymatic cell dissociation solution (Thermofisher Scientific, ref 13150016) at 37°C, washed twice in complete DMEM and incubated for 1 h at 4°C with primary antibodies in complete DMEM. After three washes, the cells were incubated for 1 h at 4°C with a phycoerythrin‐conjugated F(ab')_2_ goat anti‐mouse antibody (Jackson ImmunoResearch). The cells were analysed using a Cytoflex S flow cytometer (Beckman Coulter). The geometric mean fluorescence intensity was used for quantification.

### 2.4 Immunofluorescence and confocal microscopy

The cells grown on coverslips in complete medium for 2 days were washed twice in PBS, fixed for 15 min with 4% paraformaldehyde at room temperature, washed in PBS and then incubated for 15 min in 50 mM NH4Cl in PBS. The cells were then incubated for 1 h with anti‐tetraspanin antibodies in PBS supplemented with 0.1% BSA and 0.1% saponin at room temperature. The binding of primary antibodies was revealed using appropriate anti mouse IgG subclasses antibodies coupled to Alexa Fluor 488, 568 or 647 (Thermo Scientific). The cells were mounted in Vectashield Vibrance (Vector) supplemented with DAPI and examined with a Leica SP5 confocal microscope (63× objective, 1.4 numerical aperture, zoom 3). The antibodies used were TS9 (IgG1), TS81 (IgG2a) and an isotype‐switched variant of TS63, TS63b (IgG2b, Charrin et al., 2020). Quantification of the relative CD9 and CD81 signals was performed using a protocol developed in Icy imaging software (de Chaumont et al., 2012) (http://icy.bioimageanalysis.org). For differential labelling of the surface and intracellular pools of CD9P‐1 and EWI‐2, the cells were first labelled with primary and secondary antibodies in the absence of saponin, to label the surface pool. This procedure was performed again in the presence of 0.1% saponin and with a secondary antibody coupled to a different fluorochrome to label the intracellular pool (and the surface pool again).

### 2.5 EV isolation by size exclusion chromatography (SEC)

**FIGURE 1:**
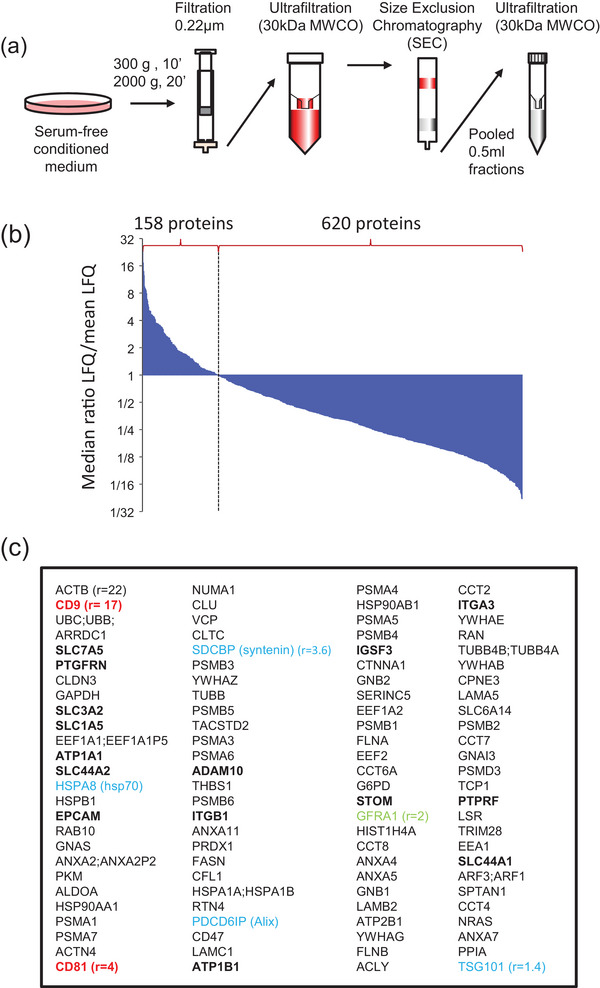
Proteomic analysis of extracellular vesicles secreted by MCF7 cells. (a) Schematic representation of the protocol used to collect EVs from the conditioned medium of MCF7 cells cultured for 16 h in the absence of serum. (b) Distribution of the proteins identified in EVs according to their relative LFQ Value. After selection of the proteins identified and quantified in at least four experiments, the LFQ value of each protein was divided by the mean LFQ value of the corresponding experiment. The graph shows the distribution of the median value of the ratio calculated in the six experiments. (c) Top proteins identified in the EVs released by WT MCF7 cells according to the median of the relative LFQ values in six experiments. Classical EVs markers are in blue. Proteins in bold characters correspond to proteins previously shown to be co‐immunoprecipitated with tetraspanins. The median LFQ ratio of some proteins is shown.

MCF7 cells were seeded in two 150 cm^2^ flasks (5 × 10^6^ cells/flask) and cultured in complete medium for 4 days, when they reach sub‐confluency. The medium was then replaced by serum‐free DMEM medium and the cells were further cultured for 16 h. The cell culture supernatant was collected for EV isolation and the cells detached and counted using trypan blue exclusion. The viability was >95%. The culture medium was subjected to centrifugation at 4°C at 300 × *g* for 10 min to pellet floating or dead cells and then at 2000 × *g* for 20 min to remove large particles. It was then filtered through a 0.22 um filter and concentrated to a volume of 500 μL by ultrafiltration using Vivas pin 20 centrifugal concentrators with a 30 kDa membrane (Sartorius). The concentrated medium was loaded on a 35 nm SEC column (IZON). Following the manufacturer's instructions, 500 μL fractions were collected and fractions 7–10 highly enriched in vesicles were pooled and further concentrated to 50 μL using ultrafiltration (Figure 1a).

### 2.6 Nanoparticle tracking analysis (NTA)

The concentration of EVs was determined using the ZetaView PMX‐120, at ×10 magnification, with software version 8.05.02. The instrument settings were 22°C, gain of 26 and shutter of 70. Measurements were done at 11 different positions (two cycles per position) and frame rate of 30 frames per second. Image evaluation was done on particles with minimum brightness: 20, minimum area: 10, maximum area: 500, maximum brightness: 255. Tracking radius two was 100, and minimum tracelength: 7.

### 2.7 Western‐blot and immunoprecipitation

After washing in PBS and detachment by scrapping, the cells were lysed for 30 min in a lysis buffer containing 30 mM Tris pH 7.4, 150 mM NaCl, 1% Triton X‐100 and a protease inhibitor cocktail (From Sigma Aldrich, diluted 200 times). The insoluble material was removed by centrifugation at 12,000 × *g* for 15 min at 4°C and the supernatant was mixed with a 3× concentrated Laemmli sample buffer. The proteins in cell and EV lysates (corresponding to 1/120 and 1/5 of a 150 cm^2^ flask respectively) were separated by SDS‐polyacrylamide gel electrophoresis and transferred to a PVDF membrane. They were visualized using the Odyssey Infrared Imaging System (LI‐COR Biosciences) after incubation of the membrane with appropriate combinations of primary and fluorescent secondary antibodies. Because all quantifications are relative, we chose not to normalize the different samples.

Immunoprecipitations were performed as previously described (Charrin et al., 2001) after biotin‐labelling of surface proteins with 0.5 mg/mL EZ‐link‐Sulfo‐NHS‐LC‐biotin (ThermoFisher scientific) for 35 min at 4°C. After lysis as above, the cell lysate was precleared for 2 h by addition of heat‐inactivated goat serum pre‐adsorbed on protein G sepharose beads (GE Healthcare 1 μL goat serum/20 μL beads/mL cell lysate). Proteins were then immunoprecipitated for 2 h using 0.4 μL of ascitic fluid mAb and 10 μL protein G‐sepharose beads for 350 μL of the lysate. The immunoprecipitated proteins were visualized using Alexa‐680‐labelled streptavidin. The same membrane was probed with the anti‐EWI‐2 mAb which was revealed using a secondary antibody coupled to Dylight 800 (Thermo Scientific).

### 2.8 Proteomic analysis

The protein extracts were stacked inside 3–4 mm of 10% SDS‐PAGE gels and stained with Imperial Stain Coomassie blue (Pierce). Each short protein lane was excised in one unique piece of gel and sliced into small pieces (about 1 mm^3^) for further in‐gel digestion with trypsin. Samples were destained using 50% ethanol in 50 mM NH_4_HCO_3_ (Sigma) at 60°C, reduced with 10 mM DTT (GE Healthcare) in 50 mM NH_4_HCO_3_ at 56°C and alkylated by adding 50 mM iodoacetamide (Sigma) in 50 mM NH_4_HCO_3_ in the dark, RT. Digestion was performed overnight at 37°C with 400 ng of trypsin (Promega) in 50 mM NH_4_HCO_3_, 5% acetonitrile solution at pH 8. Supernatants were collected and the gel pieces were rinsed twice with 0.1% trifluoroacetic acid (Sigma) and 60% acetonitrile in an ultrasonic bath to extract residual peptides. Peptides were dried using a vacuum centrifuge and resuspended in 0.1% formic acid (Sigma) and 2% acetonitrile. Automated peptide desalting was performed with the DigestPro MSi station (Intavis) using home‐made StageTips (three layers of 3 M Empore C18 Disc stacked inside a 10 μL tip) as described in Hamada et al. (2021).

Peptides were analysed with a nanoElute UHPLC (Bruker) coupled to a timsTOF Pro mass spectrometer (Bruker). Peptides were directly loaded and separated on an Aurora2 RP‐C18 analytical column (25 cm, 75 μm i.d., 120 Å, 1.6 μm from IonOpticks) at a flow rate of 400 nL/min with a 30 min elution gradient from 2% to 33% acetonitrile with 0.1% formic acid (from 2% to 5% in 1 min, 5% to 17% in 17 min, 17% to 25% in 7 min, 25% to 33% in 5 min). Accumulation time was set to 100 ms in the TIMS tunnel. Mass range was set from 100 to 1700 *m*/*z* in MS and MS/MS and mobility range from 0.6 to 1.6 Vs/cm^2^. Dynamic exclusion was activated for ions within 0.015 *m*/*z* and 0.015 V s/cm^2^ and released after 0,4 min. Exclusion was reconsidered if precursor ion intensity was four times superior. Low abundance precursors below the target value of 20,000 a.u. and intensity of 2500 a.u. were selected several times for PASEF‐MS/MS until the target value was reached. Parent ion selection was achieved by using a two‐dimensional *m*/*z* and 1/K0 selection area filter allowing the exclusion of singly charged ions. Total cycle time was 1.1 s with 10 PASEF cycles. The mass spectrometry proteomics data have been deposited to the ProteomeXchange Consortium via the PRIDE (Perez‐Riverol et al., 2022) partner repository with the dataset identifier PXD042940 at https://doi.org/10.6019/PXD042940.

### 2.9 Quantitative data analysis for proteomics

Bruker raw files were analysed using MaxQuant software v1.6.17.0 (Tyanova et al., 2016) against the UniProtKB human proteome database (UP000005640, 74823 entries, release 2020_02). The following parameters were used for peptides and proteins identification: trypsin as enzyme specificity with a maximum of two missed cleavages; cysteine carbamidomethylation as a fixed modification and protein N‐terminal acetylation and methionine oxidation as variable modifications; peptides minimum length of seven amino acids; precursor mass tolerance of 25 ppm (first search) and 10 ppm (main search); fragment mass tolerance of 25 ppm; 1% FDR. Label‐free protein quantification (LFQ) was performed using ‘unique+razor’ peptides with the ‘min ratio count’ parameter set to 2. The MaxQuant features ‘match between runs’ (2 min time windows and 0.05 mobility windows) and ‘normalization’ were enabled.

### 2.10 Data analysis

Each experiment included a WT sample as a reference and was analysed separately using Maxquant. The results were combined using Microsoft Access and transferred to Microsoft Excel. To analyse the protein composition of EVs released by WT cells, only the proteins identified and quantified in at least four out of six experiments were considered. To compare the LFQ values of WT samples between different experiments, the LFQ value obtained for each protein was divided by the mean LFQ value of all proteins identified in the same experiment. The median value was used to compare different proteins.

We used ‘linear models for microarray data’ (limma, (Ritchie et al., 2015)) to compare the expression levels, after log2 transformation, observed in WT and KO samples, taking into account the paired designs. Missing values were first imputed using the nearest‐neighbour approach (knn.impute from R package impute). We computed adjusted P‐values using the Benjamini‐Hochberg approach controlling the FDR at level 5%.

Funrich (Pathan et al., 2017) v3.1.3 was used to compare the listing of identified proteins with proteins previously found in EVs secreted by breast cancer cells, notably MCF7 cells. To this end, the Vesiclepedia database (Pathan et al., 2019) of vesicular proteins was downloaded (Version 4.1, August 15 2018) and filtered to retain the data from studies of breast cells. We also compared our results with those reported in two previous studies analysing the protein repertoire of EVs secreted by MCF7 cells (Hurwitz et al., 2016; Rontogianni et al., 2019). Funrich and The Database for Annotation, Visualization, and Integrated Discovery (DAVID, v2021) were used for cellular compartment and functional (GOTERM_BP_DIRECT) analyses of the proteins identified, respectively.

## 3 RESULTS

### 3.1 Protein composition of EVs released by MCF7 cells

EVs secreted by MCF7 cells were purified by SEC (Figure 1a) and their protein composition was analysed using label‐free quantitative liquid chromatography–tandem mass spectrometry (LC–MS/MS). About 1200 proteins were identified and quantified (provided that at least two peptides corresponding to this protein were identified) in at least three out of six experiments in the EV fractions purified from WT MCF7 cells, and among them 778 were identified in four out of six experiments (Figure 1b, Supplementary Table). Most of these proteins were present in EVs at a low level, as approximately 2/3 of them had a median relative ‘label free quantification’ (LFQ) value (calculated as the LFQ of the protein in one experiment divided by the average of all LFQ values in the same experiment) below 0.5 (Figure 1b, Supplementary Table). Most of them have been reported in the Vesiclepedia compendium of extracellular vesicle molecular data, and have been identified in previous mass‐spectrometry analyses of EVs secreted by MCF7 cells isolated by ultracentrifugation (Rontogianni et al., 2019) or a combination of polyethylene glycol precipitation and ultracentrifugation (Hurwitz et al., 2016) (Figure S1A). Accordingly, cellular component analysis showed that ∼70% of the proteins identified in at least 4 experiments are associated with the term ‘exosome’ (note that this term is used by the GO database as homonymous to small EVs, and not to MVB‐derived exosomes). Approximatively 70% of the identified proteins were cytoplasmic and ∼50% were associated with the term lysosome (Figure S1B). Among the proteins identified are classical markers of extracellular vesicles, including CD9, CD81 and CD63, as well as classical markers of exosomes such as syntenin (SDCBP), Alix (PDCD6IP), and Tsg101 (Figure 1c, Supplementary Table). We thus concluded that this approach is suitable for the isolation of EVs bearing tetraspanins including exosomes, although EVs not expressing these tetraspanins are likely to be co‐isolated as shown by the presence of RNA‐binding proteins (EEF2, EEF1A1) and common glycolytic Enzymes (GAPDH, ENO1, PKM) which are absent from vesicles bearing CD9, CD81 or CD63 (Jeppesen et al., 2019; Mathieu et al., 2021) (Figure 1c and Supplementary Table).

### 3.2 EV enrichment and subcellular localization of CD9, CD81 and CD63

**FIGURE 2:**
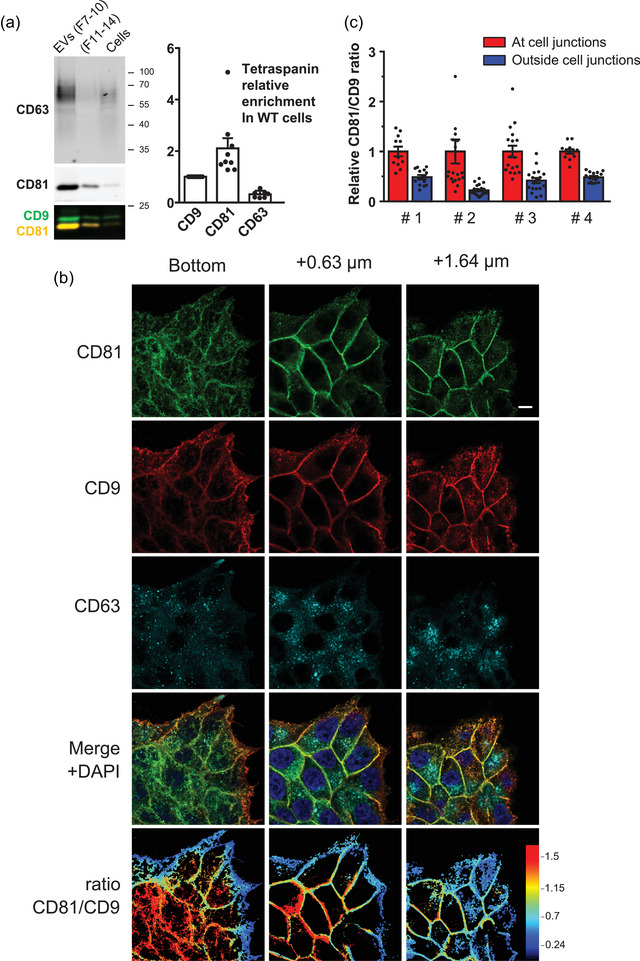
Relative enrichment of CD9, CD81 and CD63 in EVs and their subcellular distribution. (a) Western‐blot analysis of CD9, CD81 and CD63 in MCF7 lysate and in EVs they release and calculation of their enrichment in EVs. This enrichment is calculated as the signal in EVs divided by the signal in lysates, with the ratio calculated for CD9 being used as the reference. The graph shows the mean ± SEM and values of replicates. CD63 and CD81 were subsequently probed with specific antibodies and a secondary antibody coupled to Alexa Fluor 680 before incubation with the CD9 antibody and a dylight 800‐coupled secondary antibody. In this particular experiment we also analysed the pool of fractions 11–14 collected during the SEC procedure. (b) Comparison of the cellular distribution of CD9, CD81 and CD63 in MCF7 cells by confocal microscopy. The cells were fixed before labelling of CD9, CD81 and CD63 as described in material in methods. Three different confocal sections are shown. The lower images correspond to the ratio of CD81 to CD9 intensity in each pixel. Bar: 10 μm. (c) Relative levels of CD81 and CD9 at cell‐cell contacts or outside these contacts. Regions of interests were drawn around cell‐cell junctions or on the top/periphery of the cells excluding these contacts. The CD81/CD9 ratio in each region was calculated and divided by the mean of the ratios calculated in the regions drawn around cell‐cell contacts. The results for four cell clusters, similar to that shown in Figure 2b are shown.

We compared the median LFQ values, which reflect the detected peptide ions signals, of CD9, CD81 and CD63 (Supplementary Table). The median relative LFQ values of CD81 and CD63 were respectively 5 and 15 times lower than that of CD9, raising the hypothesis that CD9 is more abundant in EVs than CD81 and CD63. We next analysed their relative enrichment in EVs, that is their proportion in EVs relative to their cell expression level (Figure 2a). To calculate this relative enrichment, cell lysates and EVs proteins were loaded on the same gels, separated by electrophoresis and analysed by western‐blotting. To normalize the different experiments, the ratio of the signal in EVs to that in the cell lysate was calculated and divided by the ratio calculated for CD9 in each experiment. CD63 was 3‐fold less enriched than CD9 and CD81 was in average ∼2‐fold more enriched than CD9 (median value = 1.6; Figure 2a). In one experiment we also analysed the pool of SEC fractions 11–14 (Figure 2a) which contained only a small amount of the 3 tetraspanins, in accordance to the manufacturer's indications. These different enrichments led us to compare the subcellular distributions of CD9, CD81 and CD63 by confocal microscopy. Both CD9 and CD81 were mainly localized at the plasma membrane with very little intracellular localization, in contrast to CD63 (Figure 2b). Although CD9 and CD81 strongly colocalized in MCF7 cells, a difference was that the CD81/CD9 fluorescence ratio was higher at the basal cell surface or at intercellular contacts than at the cell periphery or at the top of the cells (Figure 2b, quantified in Figure 2c). Moreover, at cell‐to‐cell contacts, this ratio decreases from the bottom to the top of the cells (Figure S2).

### 3.3 Comparison of EVs secreted by MCF7 lacking or not CD9, CD81 or CD63

**FIGURE 3:**
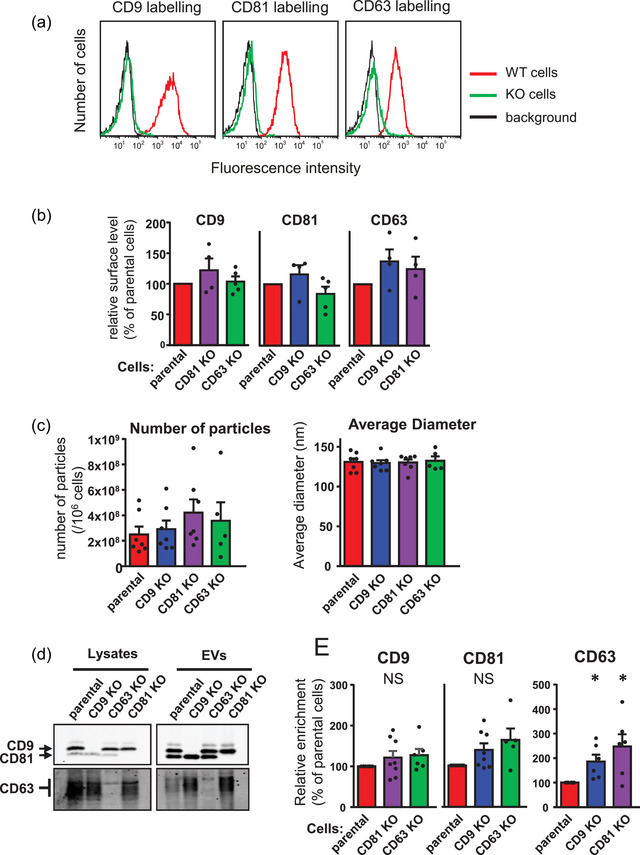
characterization of MCF7 cells lacking CD9, CD81 or CD63 and the EVs they release. (a) Flow‐cytometry analysis of the surface expression of CD9, CD81 and CD63 in parental MCF7 cells and cells KO for the corresponding tetraspanin. (b) Relative expression of the indicated tetraspanin in WT cells and cells not expressing the two other tetraspanins, as determined by flow‐cytometry. (c) Number and size of particles recovered from parental and KO cells, determined using nano‐particle tracking. The graphs show the mean ± SEM and individual biological replicates. (d) Western‐blot analysis of CD9, CD81 and CD63 in cell lysates and EVs. The lysates and EV samples were loaded on the same gel and are shown using the same settings. (e) Relative enrichment of CD9, CD81 and CD63 in the various cell lines, calculated as the signal in EVs divided by the signal in lysates, with parental cells serving as the reference. The graph shows the mean ± SEM and individual biological replicates. The difference between parental and KO cells was statistically analysed using a ratio paired *t*‐test. *, *p* < 0.05.

We generated MCF7 cells lacking CD9, CD81 or CD63 using the CRISPR/Cas9 technology. To avoid clone effects, we isolated populations of cells lacking these tetraspanins using cell sorting. Flow cytometry and western‐blot analyses confirmed that after sorting, the cells lacked the targeted tetraspanins (Figure 3a,d). There were no significant changes in the expression levels of the other tetraspanins at the cell surface, as determined by flow cytometry, although there was a trend towards a higher level of CD63 in CD9 and CD81 KO cells. (Figure 3b).

The number and size of EVs recovered from parental MCF7 cells and cells lacking the tetraspanins were compared by NTA. The recovery of particles was variable, ranging from 1.5 × 10^8^ to 5 × 10^8^ particles per million cells, and there were no significant differences between WT and KO cells (Figure 3c). Consistent with the purification procedure, only small particles with an average diameter of ∼130 nm were recovered from WT cells, which was not significantly different in the three KO cell populations (Figure 3c).

To obtain a systematic comparison of the protein composition of EVs released in the absence of one of the three tetraspanins by quantitative mass spectrometry analysis, EVs from KO cells were always prepared in parallel with those of WT cells, and the variations were statistically analysed using limma taking the paired design into account. Four experiments were performed for CD63 KO cells and six for CD9 and CD81 KO cells. The analysis was limited to the proteins most reproducibly identified in EVs from WT cells, that is in at least three out of four experiments for CD63 KO cells (596 proteins) and five out of six experiments (507 proteins) for CD9 and CD81 KO cells.

**FIGURE 4:**
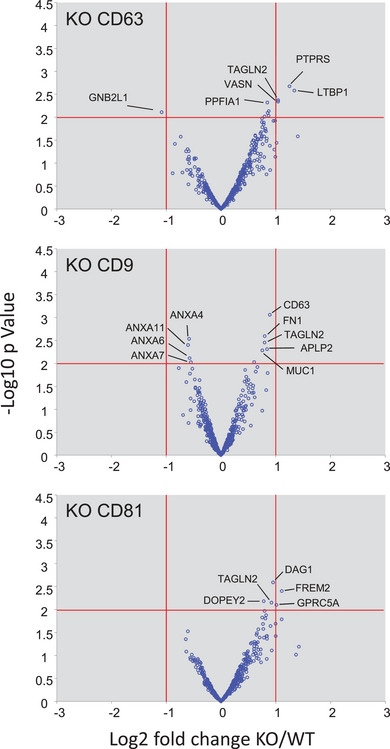
Volcano plots showing the relative expression of the proteins identified in the EVs released by parental and the different MCF7 KO cells. The changes in protein expression (expressed as the Log2 (fold change KO/WT)) are shown according to the statistical significance of the observed variations (expressed as the ‐Log10 (*p*‐value)). Only proteins identified with at least two peptides in three out of four (CD63) or five out of six replicates were considered. CD63 was never detected in CD63 KO cells. CD9 and CD81 were not detected in one and four replicates, respectively. When detected, the amount of CD9 in CD9 KO EVs was in average 1.4% of the amount in WT EVs.

The proteins that showed a significant difference in the absence of CD9, CD81 or CD63 with a *p*‐value < 0.01 are shown in Table 1 and Figure 4. As shown in the Supplementary Table, none of the differences were large enough to reach significance when controlling for a False Discovery Rate of 5%. To summarize, the vast majority of proteins that showed a difference in the absence of one of the three tetraspanins varied less than 2‐fold and were generally poorly abundant in EVs as shown by the low median LFQ ratio value. However, one protein (GNB2L1) showed a significant 2‐fold reduction in EVs secreted by CD63 KO cells. Also, four proteins that belong to the Annexin family of proteins were significantly reduced (but less than 2‐fold) in EVs secreted by CD9 KO cells. Two of them, ANXA7 and ANXA11 have been shown to be present in EVs expressing CD9 and CD81 in several studies (Jeppesen et al., 2019; Kowal et al., 2016; Mathieu et al., 2019). Of note, there were no changes in ANXA2 and ANXA5 levels, which are only occasionally found in these EVs.

**TABLE 1:** List of proteins showing a significant change with a *p*‐value < 0.01. The median ratio corresponds to the median of the ratios between the LFQ value and the mean LFQ in each experiment. Note that except for the targeted tetraspanins, none of the differences observed were statistically significant after adjusting the p‐values to control for a false discovery rate of 5%. (1) CD81 was not detected in the dKO samples in one experiment. The two values correspond to the mean log2 fold change in the other three experiments and after imputation of the missing value.

		Mean log2 fold change			
Gene names	Median ratio	CD63 KO	CD9 KO	CD81 KO	dKO CD9, CD81
Tetraspanins and partners					
CD63	1.2	Not detected	0.89		1.34
CD9	18.4		−5.71		−3.94
CD81	4		0.44 (*p* = 0014)	4/6 Not detected	−3.5/−2.83^1^
PTGFRN	9.2				−1.11
IGSF8	0.98				−1.50
Decreased					
GNB2L1	1	−1.08			
ANXA11	3		−0.60		
ANXA4	1.9		−0.58		
ANXA6	1.3		−0.57		
ANXA7	1.6		−0.55		
Increased					
PTPRS	0.11	1.34			
LTBP1	0.15	1.25			
VASN	0.11	1.04			
TAGLN2	0.11	1.04	0.80	0.92	
PSMA2	0.98	0.87			
NRCAM	0.23	0.87			
PPFIA1	0.09	0.85			
UGP2	0.34	0.85			
PSMB1	2.2	0.80			
APLP2	0.13		0.84		
FN1	0.78		0.80		
MUC1	0.77		0.76		
FBP1	0.33		0.61		
FREM2	0.78			1.11	
GPRC5A	0.33			1.01	
DAG1	0.09			0.95	
DOPEY2	0.18			0.78	
TTYH3	0.56				1.66
TSPAN6	0.71				1.33
CIB1	0.16				1.18

There was no significant change in CD9 or CD81 levels in the EVs released by CD63 KO cells (Table 1), consistent with western‐blot analysis (Figure 3d,e) showing no change in CD9 or CD81 enrichment in the EVs of CD63 KO cells (expressed as the ratio of tetraspanin in EVs to cell lysate). In contrast, by mass‐spectrometry we observed an almost two‐fold increase in CD63 in the EVs produced by CD9 KO cells, and a more modest increase in CD81 (although with a p‐value of only = 0.014). These increases were consistent with the western‐blot analyses of Figure 3d,e, showing higher CD63 and CD81 enrichment in the EVs released by CD9 KO cells, although the latter was not statistically significant. By contrast, although western‐blot analysis showed a variable but statistically significant increase in CD63 enrichment in the EVs secreted by CD81 KO cells, an increase was observed in the proteomic analysis only in 3 of the 6 biological replicates and was not statistically significant.

In conclusion, the absence of CD9, CD81 or CD63 has a modest impact on the protein composition of EVs secreted by MCF7 cells, and the major change observed is a small decrease of several annexins in EVs when CD9 is absent.

### 3.4 Proteomic analysis of EVs lacking both CD9 and CD81

**FIGURE 5:**
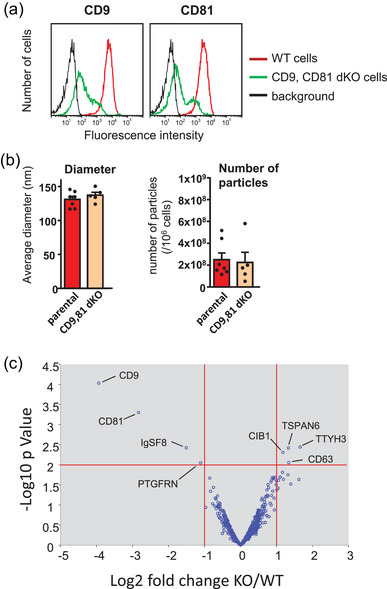
Impact of a deficiency in both CD9 and CD81 on EV production and composition. (a) Flow‐cytometry analysis of the surface expression of CD9 and CD81 in parental MCF7 cells and CD9, CD81 dKO cells. (b) Number and size of particles recovered from parental and CD9,CD81 dKO cells, using nano‐particle tracking. The graphs show the mean ± SEM and individual biological replicates. (c) Volcano plots showing the relative expression of the proteins identified in EVs released by parental MCF7 cells and CD9, CD81 dKO cells (expressed as the Log2 (fold change KO/WT)) according to the statistical significance of the observed variations (expressed as the ‐Log10 (p‐value)). Only proteins quantified with at least two peptides in three out of four replicates in parental cells were analysed.

**FIGURE 6:**
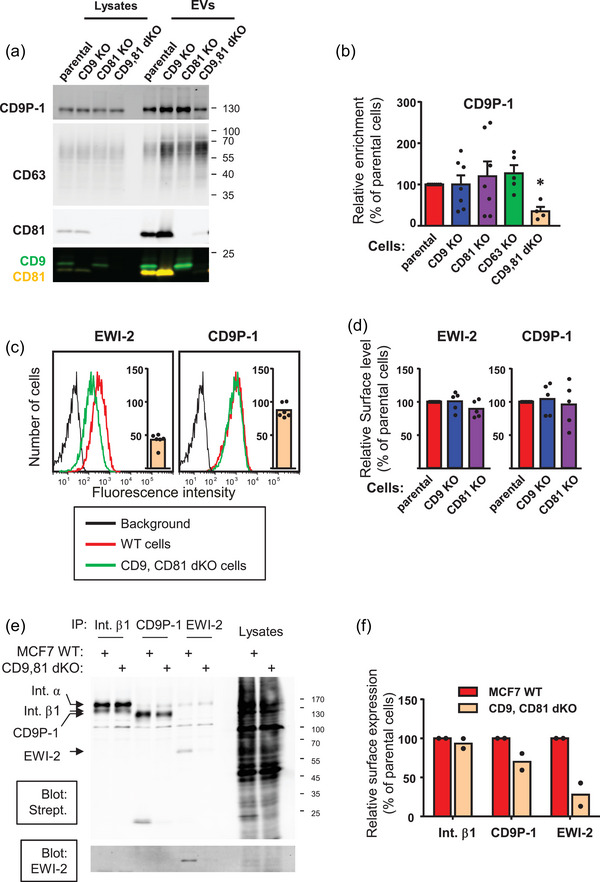
Impact of the deficiency in both CD9 and CD81 on CD9P‐1 and EWI‐2 expression levels. (a and b) Western‐blot analysis of CD9, CD81, CD63 and CD9P‐1 in cell lysates and EVs of parental and KO cells. The specific antibodies were revealed with a secondary antibody labelled with Alexa Fluor 680 except the CD9 antibody which was revealed with a dylight 800‐labeled secondary antibody. The graph shows the mean ± SEM, as well as individual biological replicates, of the relative enrichment of CD9P‐1 in the various cell types. The difference between parental and KO cells was statistically analysed using a ratio paired t‐test. *, *p* < 0.05. (c) Flow‐cytometry analysis of EWI‐2 and CD9P‐1 levels at the surface of parental and CD9, CD81 dKO cells. The graphs show the mean value of geometric mean fluorescence intensities and individual biological replicates of KO cells relative to parental cells. (d) Relative EWI‐2 and CD9P‐1 expression levels at the surface of parental, CD9 KO and CD81 KO cells, determined by flow‐cytometry. (e and f) After biotin‐labeling of surface proteins, parental and CD9, CD81 dKO cells were lysed and immunoprecipitations were performed as indicated on the top. Immunoprecipitated proteins were visualised using Alexa 680‐labelled streptavidin. The same blot was probed with the anti‐EWI‐2 mAb which was revealed using a secondary antibody coupled to Dylight 800. The graph shows the quantification of the bands corresponding to integrins, CD9P‐1 and EWI‐2 in two independent experiments, relative to the value obtained for WT samples. Int: Integrin; Strept: Streptavidin.

Beside their strong colocalization (Figure 2b), CD9 and CD81 sometimes perform similar functions, notably in the regulation of cell‐cell fusion, and both associate directly with 2 related Ig domain proteins, CD9P‐1/EWI‐F (encoded by the *PTGFRN* gene) and EWI‐2 (encoded by the *IGSF8* gene) (Charrin et al., 2014; Charrin, Le Naour et al., 2009; Hemler, 2005). We therefore generated MCF7 cells lacking both CD9 and CD81 using CRISPR/Cas9, and selected CD9 and CD81‐negative/low cells by cell sorting. Overall, flow‐cytometry analysis (Figure 5a) and western‐blot analysis (Figure 6a) showed that the total amount of these tetraspanins was reduced by more than 90%. By flow cytometry, we observed a minor population (∼30%) still expressing CD9 or CD81, but at a reduced level of ∼80% compared with parental cells, and a major population (∼70%) whose signal only partially overlapped that of the negative control (Figure 5a). We believe that this population does not synthesize CD9 or CD81, but that the slightly positive labelling is rather due to a transfer from the minor fraction of cells still expressing a residual level of these molecules (see Figure S3 for more details).

Like single CD9 or CD81 KO cells, the double KO cells showed no change in the diameter and number of particles secreted, (Figure 5b). A comparative quantitative proteomic analysis was performed as above on the EVs from CD9/CD81 double KO cells, isolated in parallel with those of WT cells (Figure 5c). Surprisingly, the proteins that were increased in the EVs of single CD9 or CD81 KO cells were not increased in the EVs of dKO cells, except for CD63 (also confirmed by western‐blot, Figure 6a). Moreover, the decrease in several Annexins observed in the EVs of CD9 KO cells was not recapitulated in the EVs of CD9/CD81 dKO cells. Potentially interesting because of its ability to regulate exosome formation and composition (Andrijes et al., 2021; Ghossoub et al., 2020; Guix et al., 2017) is the >2‐fold increase in Tspan6 levels (Figure 5c, Table 1).

The major changes observed in the EVs released by CD9/CD81 dKO MCF7 cells were the >2 fold reduction (up to 75% reduction) of CD9P‐1/PTGFRN and EWI‐2/IGSF8. Western‐blot analysis showed a lower targeting of CD9P‐1 in the EVs of CD9/CD81 dKO cells (Figure 6a, quantified in Figure 6b). We could not analyse the expression of EWI‐2 by western‐blot because the expression of EWI‐2 was too low in this cell line and/or the only mAb available not efficient enough in this application.

### 3.5 Differential regulation of CD9P‐1/PTGFRN and EWI‐2/IGSF8 expression levels by CD9 and CD81

Flow‐cytometry analysis showed a ∼50% decrease of EWI‐2 at the surface of CD9/CD81 dKO cells (Figure 6c). Immunoprecipitation of EWI‐2 after biotin labelling of cell surface proteins confirmed this diminished EWI‐2 surface expression (∼70% reduction, mean of two experiments, Figure 6e, quantified in Figure 6f). Immuno‐blotting of these immunoprecipitates using the anti‐EWI‐2 mAb showed that the total level of EWI‐2 was reduced in these cells (Figure 6c). Quantitative RT‐qPCR showed no change of EWI‐2 mRNA levels (data not shown). In contrast, there was only a mild decrease in CD9P‐1 surface expression (12% reduction as determined by flow‐cytometry, 30% reduction as determined by immunoprecipitation, (Figure 6c,e,f) and no change at the mRNA level (data not shown)). A decrease of the same magnitude of total CD9P‐1 expression level was observed by western‐blot (∼25%, Figure S4A,B). As a control, there was no change in β1 integrins surface levels (Figure 6e,f). Analysis of two clones completely lacking CD9 and CD81 (Figure S4C,D) confirmed that the surface expression level of EWI‐2 was more dependent on the expression of these tetraspanins than that of CD9P‐1, although both proteins were still present at the plasma membrane to some extent in the complete absence of the two tetraspanins. Importantly, there was no change of CD9P‐1 or EWI‐2 surface levels in single CD9 or CD81 KO cells (Figure 6d).

Confocal microscopy analysis showed that CD9P‐1 was mainly localized at the cell surface in parental cells. This analysis confirmed the decrease in CD9P‐1 surface expression in the two clones of CD9, CD81 dKO cells and showed no major intracellular accumulation of the protein inside the cells (Figure S5A). Because endogenous EWI‐2 was barely detectable using this approach, we analysed the subcellular distribution of this protein after transfection. This protein was mainly expressed at cell surface, in both parental and CD9, CD81 dKO cells (Figure S5B). However, if there was some intracellular staining in parental cells, that of dKO cells was characterized by a dot‐like pattern (Figure S5B). Thus, the subcellular trafficking of transfected EWI‐2 is different in parental and CD9, CD81 dKO cells. There was no overlap between EWI‐2 and SEC61β  (Figure S5C), an Endoplasmic Reticulum marker, indicating that after transfection EWI‐2 efficiently exits from the ER in both parental and dKO cells.

## 4 DISCUSSION

In this study, using proteomic approaches we investigated whether the three tetraspanins CD9, CD81 and CD63, which are among the most widely used markers of EVs, regulate the sorting of cargo proteins into EVs. Very few proteins showed reproducible changes upon deletion of these tetraspanins. However, down‐regulation of both CD9 and CD81 together was associated with a decrease of CD9P‐1/EWI‐2/PTGFRN and EWI‐2/IgSF8 in EVs.

### 4.1 A tetraspanin view of EV composition

The protein composition of EVs reported here may to some extent help to understand the origin of these EVs. Among the most abundant proteins identified in EVs were proteins recently shown to be mostly present, in HeLa cells, on CD9‐positive vesicles released from the plasma membrane, such as CD9P‐1 (PTGFRN) and the 4F2/LAT1 complex (SLC3A2/SLC7A5) (Mathieu et al., 2021) (Figure 1c and Supplementary Table) as well as ARRDC1, specific of a category of small plasma membrane‐derived EVs (Nabhan et al., 2012). In addition, the peptides corresponding to CD9 were the most abundant after those of actin, while the abundance of CD63 peptides, as determined by the relative LFQ median value, was 25 times lower (Table 1 and Supplementary Table). Although we cannot exclude that this lower LFQ value for CD63 is due to a poor detection of the corresponding peptides, western‐blot analysis showed that CD63 was less enriched than CD9 in EVs (i.e. the EV to cell level ratio is lower), similarly to other cell lines (Fordjour et al., 2022; Mathieu et al., 2021). Altogether these data are compatible with the hypothesis that in MCF7 cells, like in HeLa and HEK 293 cells (Fordjour et al., 2022; Mathieu et al., 2021), EVs bearing CD63 and formed in late endosomes (i.e. bona fide exosomes) are a minor population, whereas EVs bearing CD9 formed at the plasma membrane (i.e. ectosomes) are a major sub‐population of EVs. Further work will be required to validate this hypothesis.

Western‐blot analysis showed that in the MCF7 cell line CD81 was on average ∼2‐fold more enriched in EVs than CD9 (Figure 2a). Again, a higher enrichment of CD81 compared to CD9 has previously been reported in other cells (Fordjour et al., 2022). This raises the hypothesis that a substantial fraction of EVs bud from regions of MCF7 cells that have a relatively higher CD81 local concentration. Both CD9 and CD81 where mainly localized at the cell surface, where they strongly colocalized, with little intracellular localization (Figure 2b). This is again consistent with the idea that the EVs they decorate originate from the cell surface, although we cannot exclude that a fraction of them comes from an intracellular compartment in which their passage is transient. Because the CD81/CD9 fluorescence ratio was higher at the basal surface of the cells or at intercellular contacts compared to the cell periphery or the top of the cells, a simple explanation for the stronger enrichment of CD81 in EVs secreted by MCF7 cells might be that a significant fraction of EVs is released from these areas. Although this may seem counter‐intuitive, cell‐cell and cell‐matrix contacts are highly dynamic, and one may hypothesize that ‘rubbing’ between cells or the interaction with the substrate favour the release of membrane fragments in the form of EVs.

In addition to CD9, CD81 and CD63, five tetraspanins were identified in these EVs, with at least two peptides in four out of six experiments (Supplementary Table): CD151, Tspan1, Tspan6, Tspan14 and Tspan15. Tspan6 has recently drawn some attention in EV biology because like CD63 it associates with syntenin (Guix et al., 2017; Latysheva et al., 2006), a key regulator of exosome formation (Baietti et al., 2012). Tspan6 has been shown to regulate the recruitment of proTGFα to exosomes in a syntenin‐dependent manner (Andrijes et al., 2021), and to regulate the formation of exosomes (Ghossoub et al., 2020; Guix et al., 2017). It should also be noted that the integrin α3β1 that directly associates with CD151 (Serru et al., 1999; Yauch et al., 1998), and the metalloprotease ADAM10, the only identified direct partner of Tspan14 and Tspan15 (Dornier et al., 2012; Haining et al., 2012) are among the most abundant proteins identified in EVs. ADAM10 has long been shown to be present and active in EVs (Stoeck et al., 2006) and recent data suggest that it constitutes a marker of small EVs, and especially of those that express CD81 (Kowal et al., 2016; Lischnig et al., 2022; Martin‐Jaular et al., 2021)

More generally, among the top 100 most abundant proteins identified in EVs from MCF7 cells, nearly all plasma membrane‐associated proteins were previously shown to co‐immunoprecipitate with CD9 or CD81 (Andre et al., 2006; Bruening et al., 2018; Jouannet et al., 2016; Kovalenko et al., 2007; Le Naour et al., 2006) (Figure 2c). This suggests that interaction with these tetraspanins may facilitate sorting into EVs. Alternatively, the mild detergents used in co‐immunoprecipitation experiments may preserve to some extent membrane “structures” or nanodomains enriched in tetraspanins at the origin of a subset of EVs.

### 4.2 CD9, CD81 and CD63 have little impact on EV protein composition

Our proteomic analysis revealed only minor changes in the protein composition of EVs released by MCF7 cells lacking CD9, CD81 or CD63. Importantly, none of the differences were large enough to reach significance when controlling for a False Discovery Rate of 5%, indicating that not all differences observed may be real.

One of the reproducible changes observed by mass‐spectrometry was an almost two‐fold increase of CD63 in the EVs produced by CD9 KO cells (and cells deficient in both CD9 and CD81) which was consistent with the western‐blot analyses showing higher CD63 enrichment in EVs in the absence of CD9, and similar to what was observed in the EVs released by CD9 KO melanoma cells (Suárez et al., 2021). The reason for the upregulation of CD63 in the absence of CD9 remains unknown. It seems unlikely that this reflects a higher proportion of exosomes (thus MVB‐derived EVs) in the EV preparation because no other exosome markers were increased. Another possible explanation is that in the absence of CD9, more CD63 localizes in areas from which plasma membrane‐derived EVs bud. In this regard, we observed in some experiments a small increase of CD63 at the surface of CD9, CD81 or CD9, CD81 dKO cells (Figure 3b and data not shown), although this was not statistically significant. We did not detect by confocal microscopy evident changes of CD63 localization in CD9, CD81 dKO cells (data not shown). However, we cannot exclude subtle localization changes not uncovered by confocal microscopy.

A few previous studies have reported changes in the EV protein composition in the absence of CD63, CD81, or CD9. One study focused on the impact of CD81 absence using lymphoblasts derived from WT and CD81 KO mice (Perez‐Hernandez et al., 2013). Because CD81 plays an important role in the immune response, and notably in the humoral response (Levy, 2014), the observed changes could be due to different subpopulations of lymphocytes. In this regard, the authors noted a decrease in B cell‐specific molecules such as CD20 or immunoglobulins. Three studies investigated the impact of CD63 KO, CD9 KO or CD9 silencing on the protein composition of EVs secreted by different cell lines (Brzozowski et al., 2018; Hurwitz et al., 2018; Suárez et al., 2021). In all three studies, the lack of the tetraspanin was associated, to some extent, with a change of the protein composition of EVs, with in all cases a set of proteins being up‐ or down‐regulated. However, there was little overlap between the two studies that addressed the role of CD9. Moreover, some of the proteins that were decreased following CD9 KO, including metabolic enzymes and proteasome subunits, were not present in tetraspanin‐decorated EVs (Jeppesen et al., 2019), suggesting an indirect effect of the absence of the tetraspanin. We also identified such membrane components in our cells, for example the glycolytic Enzymes GAPDH, ENO1, and PKM or several proteasome proteins, but their abundance in EVs was not modified in the absence of the three tetraspanins studied.

The lack of consistency between different studies argues against a general and essential role of tetraspanins in the targeting of cargo proteins to EVs. The reason why we detected fewer changes in the absence of CD9, CD81 or CD63 than in the previous studies cited above is unknown. This could be due to the way cells lacking tetraspanins were generated or to the different protocols used to isolate EVs and analyse the data. Furthermore, each study analysed a different cell line, which may show different subcellular distributions of these molecules, different compensation by other tetraspanins and a different role in EV cargo loading. In all cell lines we have tested, the bulk of CD9 was present at cell surfaces whereas that of CD63 was intracellular. In contrast, CD81 was enriched in an intracellular compartment in some of these cells (data not shown) in which it may have a role in EV cargo loading not testable in MCF7 cells. In addition, some of these studies used isobaric labelling methods (Brzozowski et al., 2018; Suárez et al., 2021) which show greater precision than the label‐free quantification method (O'Connell et al., 2018; Sivanich et al., 2022) we used in the present study. If such methods might have allowed us to detect more statistically significant changes, it is however unlikely that they would have uncovered more proteins whose targeting to EVs is greatly dependent on tetraspanins (i.e. showing a 2‐fold reduction or more in EVs released by cells lacking one of the three tetraspanins). Finally, another potential limitation of our study is the residual level of CD9 and CD81 in dKO cells, which could allow optimal targeting of certain cargoes to EVs, even though we detected a reproducible decrease of CD9P1 and EWI‐2 in EVs.

#### 4.2.1 CD9 and CD81 tightly regulate CD9P‐1 and EWI‐2 expression and/or trafficking

CD9 with CD81 are among the most closely related tetraspanins, having 45% identity and 70% similarity at the amino acid level. By comparison, they have only 22% and 21% identity with CD63. It is therefore not surprising that they have been shown to share several functions, and that they associate directly with the same molecular partners, namely the two related Ig domain proteins, CD9P‐1/EWI‐F (encoded by the PTGFRN gene) and EWI‐2 (encoded by the IGSF8 gene) (Charrin et al., 2014; Charrin, Le Naour et al., 2009; Hemler, 2005). These two proteins were the only ones to be significantly decreased by more than 50% in the EVS of MCF7 cells deficient in both CD9 and CD81 (Figure 5c, Table 1). This validates the ability of our approach to identify proteins whose targeting to EVs is highly dependent on the tetraspanins studied here and further highlights the functional relevance of these complexes.

We have shown that the deficiency of both CD9 and CD81 was associated with a decrease of EWI‐2 expression at the protein level (Figure 6c) but not at the mRNA level, which is likely to explain at least in part the decrease of EWI‐2 in EVs. Importantly, there was no change of EWI‐2 expression level (as determined by flow‐cytometry) in cells that lack only one of these two tetraspanins, indicating that both tetraspanins regulate EWI‐2 expression level. This decrease in EWI‐2 expression level is consistent with a previous finding showing that after transduction in U937 cells, which express neither CD9 nor CD81, EWI‐2 did not efficiently reach the cell surface unless CD81 was also co‐expressed (Stipp et al., 2003) and the finding that the expression level of EWI‐2 on the surface of *Cd9* ^−/−^ oocytes was <10% of the wild‐type level (He et al., 2009). Our findings further suggest that the expression level of EWI‐2, and possibly its intracellular trafficking, is tightly regulated by the tetraspanins with which it associates directly. CD81 and the six TspanC8 tetraspanins have previously been shown to regulate the exit from the Endoplasmic Reticulum of proteins to which they directly associate, respectively CD19 and ADAM10 (Dornier et al., 2012; Haining et al., 2012; Levy, 2014). In contrast, there was no accumulation of EWI‐2 in the endoplasmic Reticulum after transfection in CD9, CD81 KO cells (Figure S5C).

The decrease in CD9P‐1 in EVs released by cells deficient in both CD9 and CD81, which is of the same magnitude as that of EWI‐2, is not accompanied by a similar decrease in expression level. Moreover, western‐blot analysis showed that the proportion of CD9P‐1 targeted to EVs was decreased in these cells (Figure 6a). Thus, the targeting of at least a fraction of CD9P‐1 to EVs is likely to depend on the presence of CD9 and CD81. EV‐associated CD9P‐1 has recently attracted attention. On the one hand, it appeared to be, similarly to CD9, a marker of vesicle budding directly from the plasma membrane of HeLa cells (Mathieu et al., 2021). On the other hand, it was recently demonstrated to constitute an efficient scaffold to enable surface display and luminal loading of a wide range of molecules (Dooley et al., 2021). It will be interesting to determine whether this property of CD9P‐1 is regulated by its association with CD9 and CD81. The observation that the major part of the extracellular domain of CD9P‐1 is not required for efficient loading of proteins onto the surface of EVs is consistent with this hypothesis since the interaction of CD9P‐1 with CD9 and CD81 is mainly dependent on the transmembrane regions (Andre et al., 2009; Charrin, Yalaoui et al., 2009; Oosterheert et al., 2020)

## AUTHOR CONTRIBUTIONS

**Yé Fan**: Conceptualization; formal analysis; investigation; writing—review & editing. **Cédric Pionneau**: Formal analysis; investigation; writing—review & editing. **Federico Cocozza**: Investigation; supporting. **Pierre‐Yves Boelle**: Formal analysis. **Solenne Chardonnet**: Investigation; writing—review & editing. **Stéphanie Charrin**: Investigation; writing—review & editing. **Clotilde Thery**: Conceptualization; funding acquisition; writing—review & editing. **Pascale ZIMMERMANN** Conceptualization; funding acquisition; writing—review & editing. **Eric Rubinstein**: Conceptualization; formal analysis; funding acquisition; investigation; Supervision; writing—original draft.

## CONFLICT OF INTEREST STATEMENT

The authors declare no conflict of interest.

## Supporting information

Supporting Information

**Supplementary Figure 1: Proteomic analysis of extracellular vesicles secreted by MCF7 cells**. (A) Venn diagram of proteins identified in four out of six EVs samples secreted by WT MCF7 cells compared to the breast subset of Vesiclepedia database of EVs proteins and the list of proteins secreted by MCF7 cells in the studies of (Hurwitz et al., 2016) and (Rontogianni et al., 2019).
(B) Gene ontology enrichment analysis of the EV proteins identified using Funrich (Cellular component) and DAVID database (Functional analysis). ‘Proteasome‐mediated*’ = ‘proteasome‐mediated ubiquitin‐dependent protein catabolic process’
**Supplementary Figure 2: Relative levels of CD81 and CD9 at cell‐cell contacts according to the distance from the attachment plane**.
The cells were fixed before labelling of CD9, CD81 and CD63 as described in material in methods and confocal microscopy analysis. The z‐stacks were split into three smaller stacks with only five adjacent confocal planes (starting at the fifth *z* plane, 0.5 μm above the attachment plane). Regions of interest were drawn around cell–cell junctions in each of these *z*‐stacks and the CD81/CD9 fluorescence ratios were calculated in each of the ROIs. For normalization, for each cell‐cell junction analysed the value of the middle stack was normalized to 1. The results for four cell clusters, similar to that shown in Figure 2b are shown.
**Supplementary Figure 3: Transfer of CD9 and CD81 from positive to negative cells**. The flow cytometry analysis of sorted dKO cells (Figure 5a) shows the heterogeneous levels of CD9 and CD81 in these cells, with a minor population showing a ∼80% reduction of expression levels, and a major population whose signal only partially overlaps that the negative control. We believe that this population does not synthesize CD9 or CD81, but that the labelling is rather the consequence of a transfer from the minor fraction of cells that still express a residual level of these molecules. To document the transfer between cells, parental MCF7 cells (blue), cells KO for the indicated tetraspanin (red) and a mixed culture of parental and KO cells (green) were grown for 2 days before flow‐cytometry analysis of CD9 and CD81 expression levels. As shown in Figure S3, whereas the CD9 or CD81 antibodies did not bind to the cells KO for the corresponding tetraspanin, all cells were labelled, albeit at different levels, in the mixed culture, demonstrating transfer between cells. While some of this transfer may take place via EVs, it is likely that most of it occurs during the process of cell detachment and labelling for flow‐cytometry analysis.
**Supplementary Figure 4: Expression of CD9P‐1 and EWI‐2 in the absence of CD9 and CD81**. Two clones of MCF7 cells lacking CD9 and CD81 were obtained after limiting dilution.
(A) Western‐blot analysis of CD9P‐1 and GAPDH in two independent experiments, separated with a dotted line.
(B) Relative expression of CD9P‐1 determined by western‐blot, after normalization on the amount of GAPDH in the samples.
(C) After biotin‐labelling of surface proteins, parental or two clones of cells lacking CD9 and CD81 were lysed and immunoprecipitations were performed as indicated on the top. Immunoprecipitated proteins were visualized using Alexa 680‐labelled streptavidin. The same blot was probed with the anti‐EWI‐2 mAb which was revealed using a secondary antibody coupled to Dylight 800.
(D) The graph shows the quantification of the bands corresponding to integrins, CD9P‐1 and EWI‐2 in the two clones (shown in red and blue), relative to the value obtained for WT samples. Int: Integrin; cl: clone.
**Supplementary Figure 5: Confocal microscopy analysis of the subcellular distribution of CD9P‐1 and EWI‐2 in parental and CD9,CD81 dKO cells**. (A) After fixation, the cells were incubated with a combination of mAb 1F11 to CD9P‐1 and a secondary antibody coupled to Alexa Fluor 488 to label the surface pool of CD9P‐1. A second labelling was then performed, in the presence of saponin to permeabilize the cells, with 1F11 and a secondary antibody coupled to Alexa Fluor 568.
(B) The same procedure was applied to cells transfected with a plasmid encoding EWI‐2, using the anti EWI‐2 mAb 8A12. The Alexa Fluor 568 and Alexa Fluor 488 coupled secondary antibodies were used to label the surface pool and the total pool respectively.
(C) The cells were transfected with a plasmid coding EWI‐2 together with a plasmid encoding the ER marker SEC61β fused to mCherry. After fixation the cells were labelled with the anti‐EWI‐2 mAb in the presence of saponin.
In each panel, the images of WT cells and of a CD9, CD81 dKO clone are shown with the same settings. Bar, 10 μm.
