# Supplementary material for: Differential proteomics argues against a general role for CD9, CD81 or CD63 in the sorting of proteins into extracellular vesicles
Source: J Extracell Vesicles. 2023 Jul 31;12(8):12352. doi: 10.1002/jev2.12352 (PMC10390663; doi:10.1002/jev2.12352)
Supplement: Supplementary file 2 — Supplementary Figure 1: Proteomic analysis of extracellular vesicles secreted by MCF7 cells. (A) Venn diagram of proteins identified in four out of six EVs samples secreted by WT MCF7 cells compared to the breast subset of Vesiclepedia database of EVs proteins and the list of proteins secreted by MCF7 cells in the studies of (Hurwitz et al., 2016) and (Rontogianni et al., 2019). (B) Gene ontology enrichment analysis of the EV proteins identified using Funrich (Cellular component) and DAVID database (Functional analysis). ‘Proteasome‐mediated*’ = ‘proteasome‐mediated ubiquitin‐dependent protein catabolic process’ Supplementary Figure 2: Relative levels of CD81 and CD9 at cell‐cell contacts according to the distance from the attachment plane. The cells were fixed before labelling of CD9, CD81 and CD63 as described in material in methods and confocal microscopy analysis. The z‐stacks were split into three smaller stacks with only five adjacent confocal planes (starting at the fifth z plane, 0.5 μm above the attachment plane). Regions of interest were drawn around cell–cell junctions in each of these z‐stacks and the CD81/CD9 fluorescence ratios were calculated in each of the ROIs. For normalization, for each cell‐cell junction analysed the value of the middle stack was normalized to 1. The results for four cell clusters, similar to that shown in Figure 2b are shown. Supplementary Figure 3: Transfer of CD9 and CD81 from positive to negative cells. The flow cytometry analysis of sorted dKO cells (Figure 5a) shows the heterogeneous levels of CD9 and CD81 in these cells, with a minor population showing a ∼80% reduction of expression levels, and a major population whose signal only partially overlaps that the negative control. We believe that this population does not synthesize CD9 or CD81, but that the labelling is rather the consequence of a transfer from the minor fraction of cells that still express a residual level of these molecules. To document the transfer between c [file JEV2-12-12352-s002.pdf]

Supplementary Figure 1

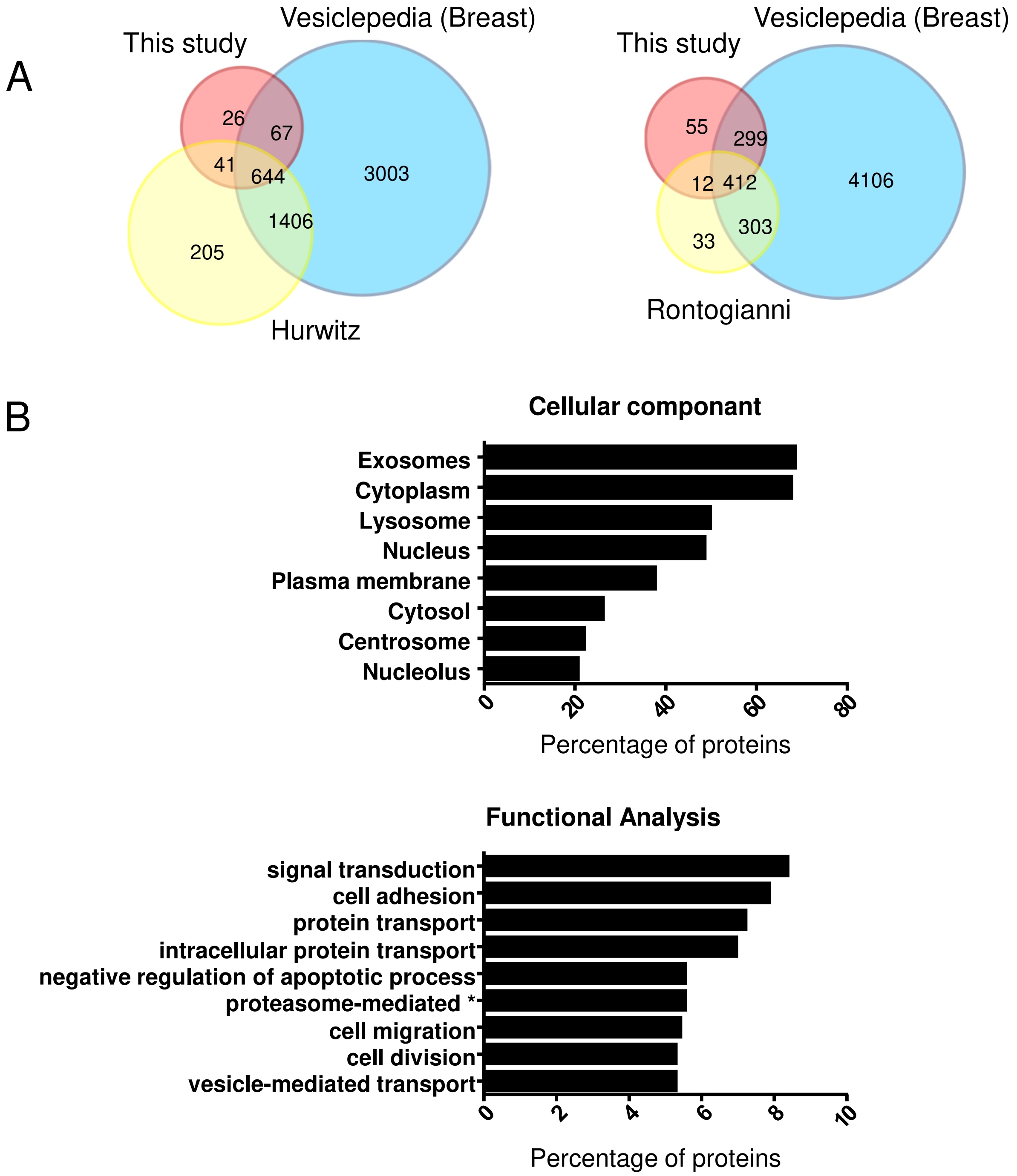

Supplementary Figure 1: Proteomic analysis of extracellular vesicles secreted by MCF7 cells.

A- Venn diagram of proteins identified in 4 out of 6 EVs samples secreted by WT MCF7 cells compared to the breast subset of Vesiclepedia database of EVs proteins and the list of proteins secreted by MCF7 cells in the studies of (Hurwitz et al., 2016) and (Rontogianni et al., 2019).
B- Gene ontology enrichment analysis of the EV proteins identified using Funrich (Cellular component) and DAVID database (Functional analysis). “Proteasome-mediated*”= “proteasome-mediated ubiquitin-dependent protein catabolic process”

Supplementary Figure 2

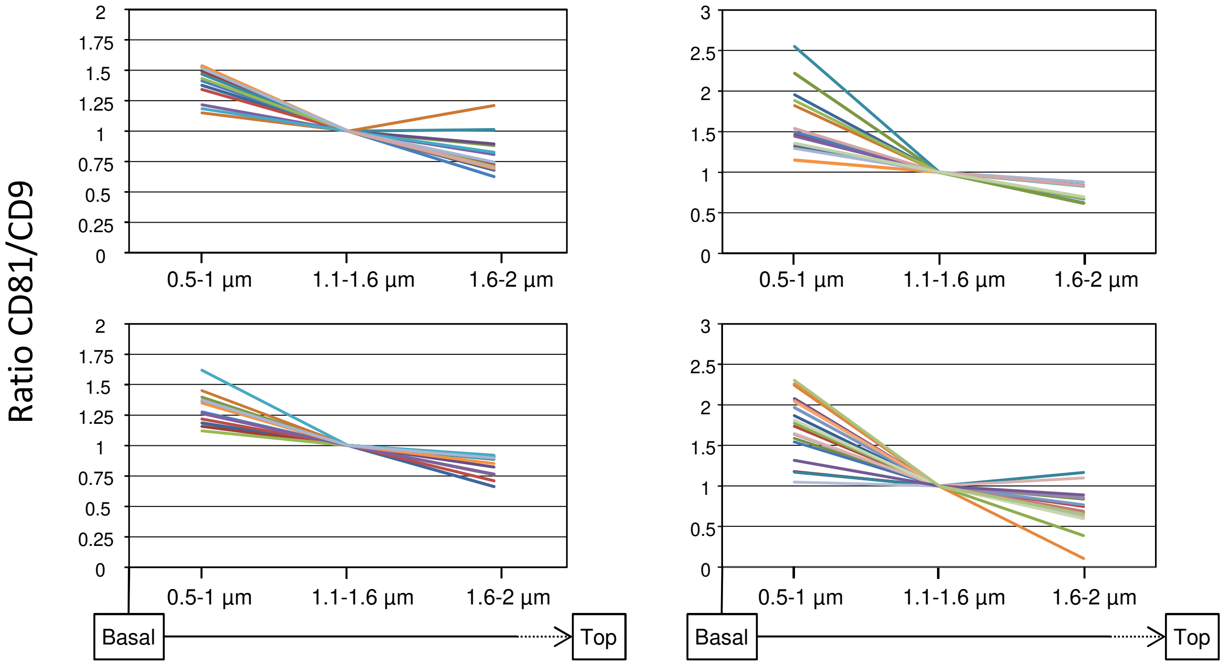

Supplementary Figure 2: Relative levels of CD81 and CD9 at cell-cell contacts according to the distance from the attachment plane.

The cells were fixed before labeling of CD9, CD81 and CD63 as described in material in methods and confocal microscopy analysis. The z-stacks were split into 3 smaller stacks with only 5 adjacent confocal planes (starting at the fifth z plane, 0.5 µm above the attachment plane). Regions of interests were drawn around cell-cell junctions in each of these z-stacks and the CD81/CD9 fluorescence ratios were calculated in each of the ROIs. For normalization, for each cell-cell junction analyzed the value of the middle stack was normalized to 1. The results for 4 cell clusters, similar to that shown in Fig.2B are shown.

Supplementary Figure 3

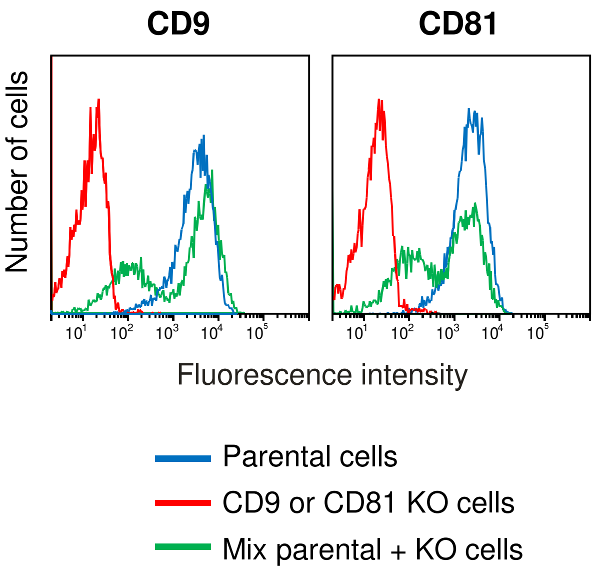

Supplementary figure 3: Transfer of CD9 and CD81 from positive to negative cells.

The flow cytometry analysis of sorted dKO cells (Fig.5A) shows the heterogeneous levels of CD9 and CD81 in these cells, with a minor population showing a ~80% reduction of expression levels, and a major population whose signal only partially overlaps that the negative control. We believe that this population does not synthesize CD9 or CD81, but that the labeling is rather the consequence of a transfer from the minor fraction of cells that still express a residual level of these molecules. To document the transfer between cells, parental MCF7 cells (blue), cells KO for the indicated tetraspanin (red) and a mixed culture of parental and KO cells (green) were grown for 2 days before flow-cytometry analysis of CD9 and CD81 expression levels. As shown in supplementary Fig.3, whereas the CD9 or CD81 antibodies did not bind to the cells KO for the corresponding tetraspanin, all cells were labeled, albeit at different levels, in the mixed culture, demonstrating transfer between cells. While some of this transfer may take place via EVs, it is likely that most of it occurs during the process of cell detachment and labeling for flow-cytometry analysis.

Supplementary Figure 4

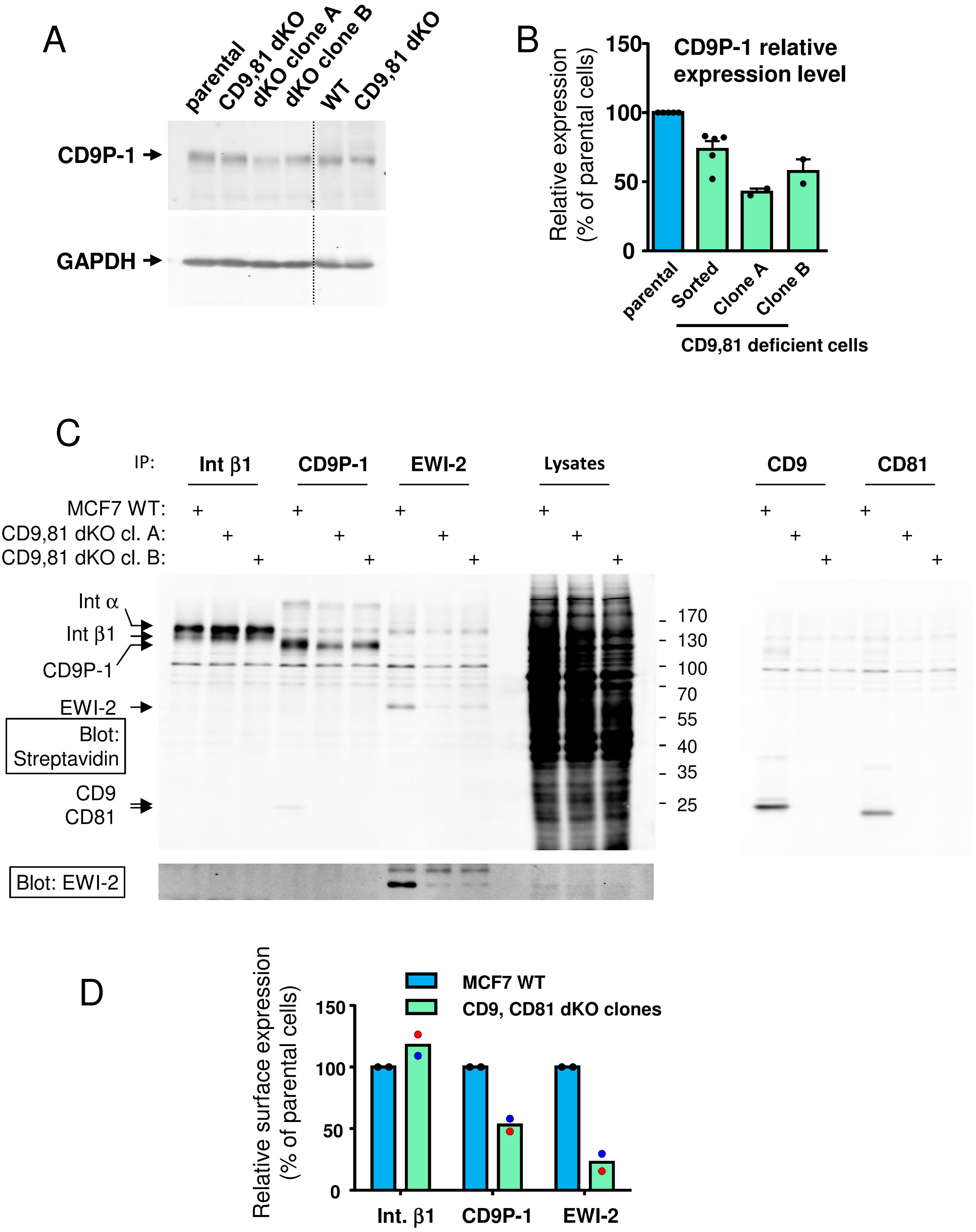

Legend on next page

Supplementary Figure 4: Expression of CD9P-1 and EWI-2 in the absence of CD9 and CD81

Two clones of MCF7 cells lacking CD9 and CD81 were obtained after limiting dilution.

A- Western-blot analysis of CD9P-1 and GAPDH in two independent experiments, separated with a dotted line.

B- Relative expression of CD9P-1 determined by western-blot, after normalization on the amount of GAPDH in the samples.

C- After biotin-labeling of surface proteins, parental or two clones of cells lacking CD9 and CD81 were lysed and immunoprecipitations were performed as indicated on the top. Immunoprecipitated proteins were visualized using Alexa 680-labelled streptavidin. The same blot was probed with the anti-EWI-2 mAb which was revealed using a secondary antibody coupled to Dylight 800.

D- The graph shows the quantification of the bands corresponding to integrins, CD9P-1 and EWI-2 in the two clones (shown in red and blue), relative to the value obtained for WT samples. Int: Integrin; cl: clone.

Supplementary Figure 5

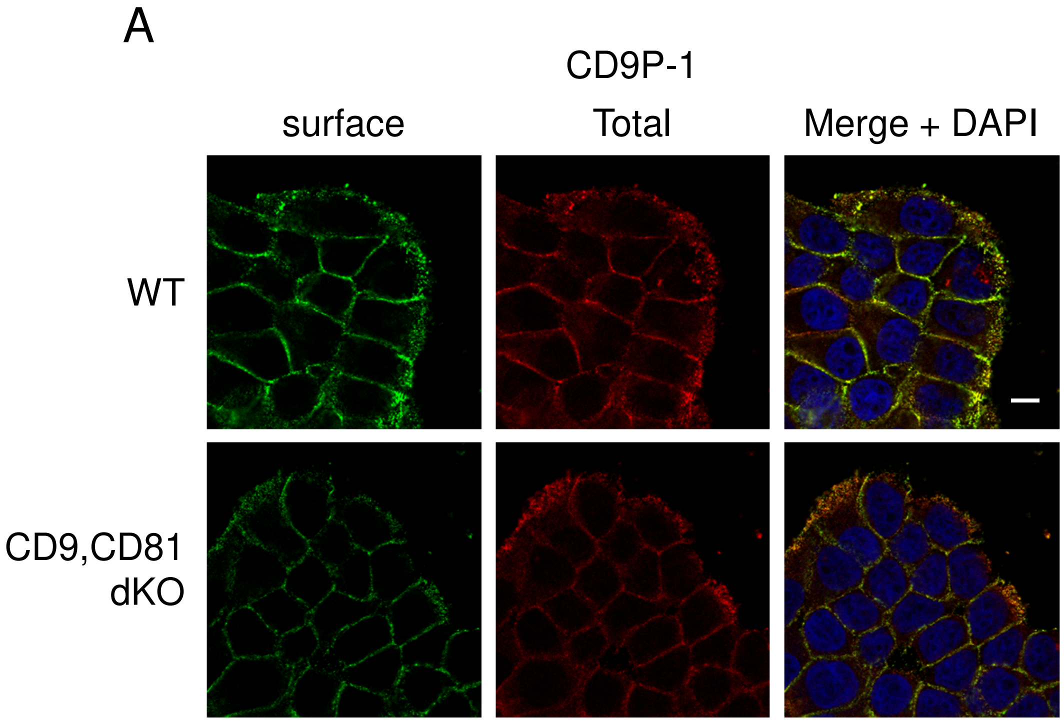

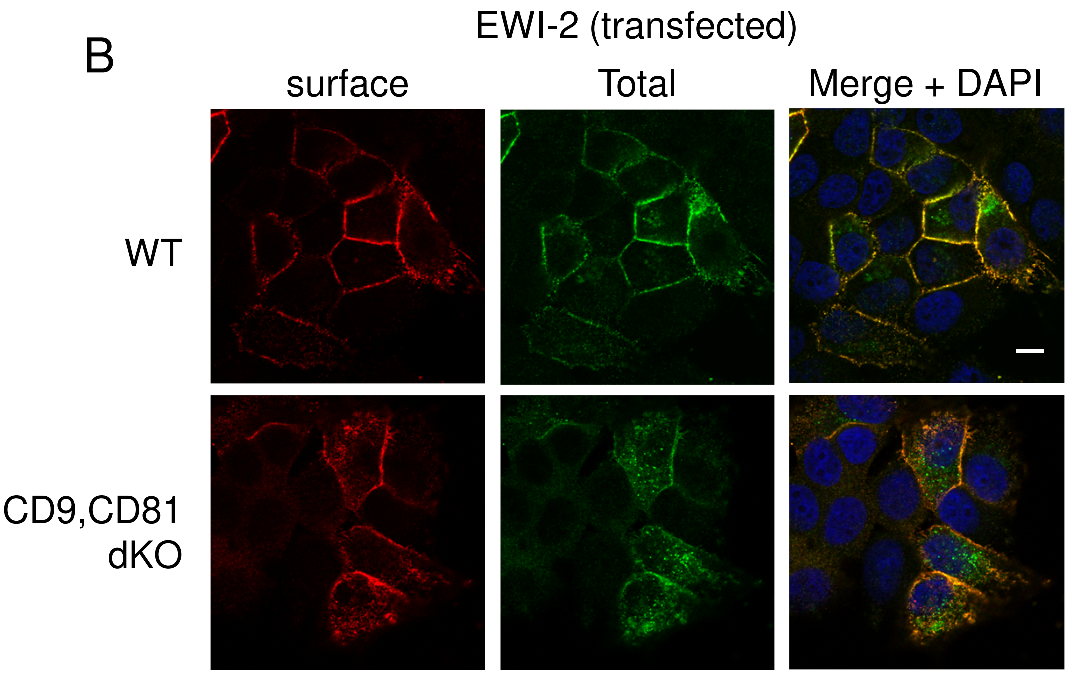

Supplementary Figure 5, continued

C

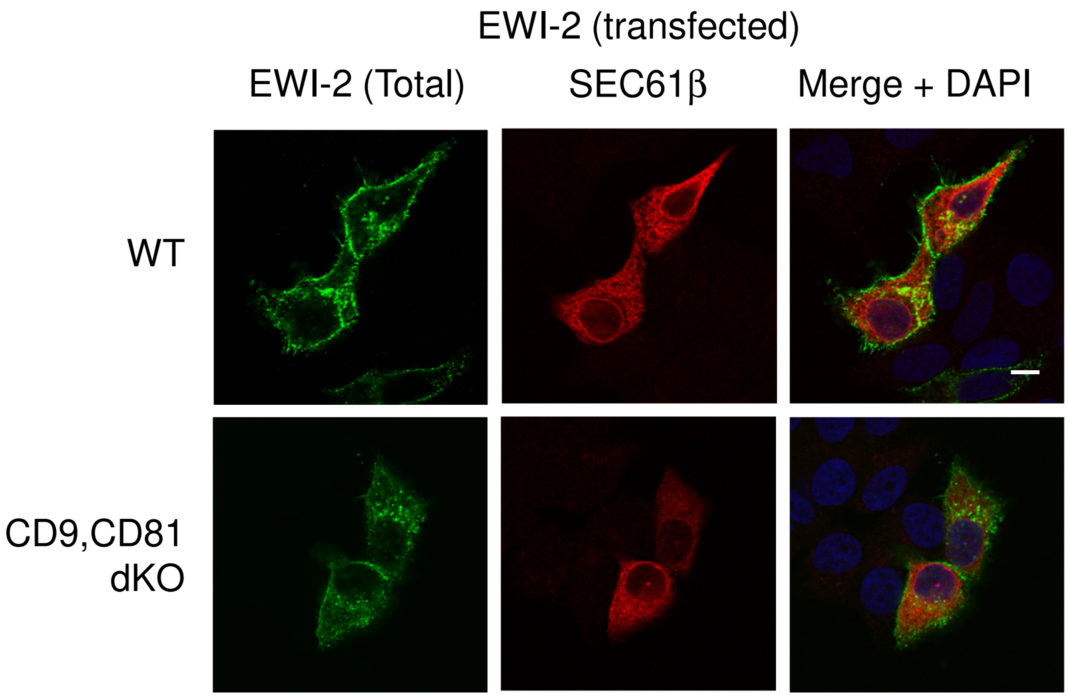

Supplementary Figure 5: Confocal microscopy analysis of the subcellular distribution of CD9P-1 and EWI-2 in parental and CD9, CD81 dKO cells.

A- After fixation, the cells were incubated with a combination of mAb 1F11 to CD9P-1 and a secondary antibody coupled to Alexa Fluor 488 to label the surface pool of CD9P-1. A second labeling was then performed, in the presence of saponin to permeabilize the cells, with 1F11 and a secondary antibody coupled to Alexa Fluor 568.

B- The same procedure was applied to cells transfected with a plasmid encoding EWI-2, using the anti EWI-2 mAb 8A12. The Alexa Fluor 568 and Alexa Fluor 488 coupled secondary antibodies were used to label the surface pool and the total pool respectively.

C- The cells were transfected with a plasmid coding EWI-2 together with a plasmid encoding the ER marker SEC61β fused to mCherry. After fixation the cells were labelled with the anti-EWI-2 mAb in the presence of saponin.
In each panel, the images of WT cells and of a CD9,CD81 dKO clone are shown with the same settings. Bar, 10 µm.
